# Complete or partial reduction of the *Met* receptor tyrosine kinase in distinct circuits differentially impacts mouse behavior

**DOI:** 10.1186/s11689-015-9131-8

**Published:** 2015-11-01

**Authors:** Barbara L. Thompson, Pat Levitt

**Affiliations:** Chan Division of Occupational Science and Occupational Therapy, Herman Ostrow School of Dentistry, University of Southern California, Los Angeles, CA 90089 USA; Institute for the Developing Mind, Children’s Hospital of Los Angeles, Keck School of Medicine, University of Southern California, Los Angeles, CA 90027 USA; Department of Pediatrics, Children’s Hospital of Los Angeles, Keck School of Medicine, University of Southern California, Los Angeles, CA 90027 USA

**Keywords:** Gene dose, Autism, Mouse, Fear learning, Behavior, Phenotype, Met

## Abstract

**Background:**

Our laboratory discovered that the gene encoding the receptor tyrosine kinase, *MET*, contributes to autism risk. Expression of *MET* is reduced in human postmortem temporal lobe in autism and Rett Syndrome. Subsequent studies revealed a role for *MET* in human and mouse functional and structural cortical connectivity. To further understand the contribution of Met to brain development and its impact on behavior, we generated two conditional mouse lines in which *Met* is deleted from select populations of central nervous system neurons. Mice were then tested to determine the consequences of disrupting *Met* expression.

**Methods:**

Mating of *Emx1*^*cre*^ and *Met*^*fx*/*fx*^ mice eliminates receptor signaling from all cells arising from the dorsal pallium. *Met*^*fx*/*fx*^ and *Nestin*^*cre*^ crosses result in receptor signaling elimination from all neural cells. Behavioral tests were performed to assess cognitive, emotional, and social impairments that are observed in multiple neurodevelopmental disorders and that are in part subserved by circuits that express Met.

**Results:**

*Met*^*fx*/*fx*^/*Emx1*^*cre*^ null mice displayed significant hypoactivity in the activity chamber and in the T-maze despite superior performance on the rotarod. Additionally, these animals showed a deficit in spontaneous alternation. Surprisingly, *Met*^*fx*/*fx; fx*/*+*^/*Nestin*^*cre*^ null and heterozygous mice exhibited deficits in contextual fear conditioning, and *Met*^*fx*/*+*^/*Nestin*^*cre*^ heterozygous mice spent less time in the closed arms of the elevated plus maze.

**Conclusions:**

These data suggest a complex contribution of Met in the development of circuits mediating social, emotional, and cognitive behavior. The impact of disrupting developmental Met expression is dependent upon circuit-specific deletion patterns and levels of receptor activity.

**Electronic supplementary material:**

The online version of this article (doi:10.1186/s11689-015-9131-8) contains supplementary material, which is available to authorized users.

## Background

*Met* encodes a tyrosine receptor kinase whose only known ligand is hepatocyte growth factor [1, 2]. MET signals through canonical ERK and PI3 kinase pathways, regulating neuronal development, including peripheral axon guidance, neuronal growth, and synapse development [3–8]. MET is expressed in excitatory projection neurons in a spatially and temporally limited pattern in the developing primate and rodent neocortex, hippocampus, and select amygdala and septal nuclei [7, 9–13]. There is conserved enrichment in growing axons and at developing synapses [9, 10], with expression decreasing dramatically during the period of pruning in primate and mouse. The specific patterns of neocortical expression, however, differ substantially between primate and rodent [10, 13, 14], suggesting possible differences in the behavioral impact of disrupted *MET* expression between species. A genetic reduction or elimination of *Met* increases local intralaminar excitatory drive in the mouse cerebral cortex [15] and disrupts the timing of excitatory synapse maturation in CA1 neurons in the hippocampus [8].

Several lines of evidence have implicated *MET* in impacting human brain function and growth. Genetic studies have demonstrated that a 5′ promoter polymorphism is associated with increased risk for autism spectrum disorder (ASD) [16–19]. This risk variant is functional, reducing MET transcription in both typical and ASD subjects [20–22]. In addition, a recent study identified a family pedigree with a rare, causal heterozygous mutation in *MET* that was associated with ASD or social-communication diagnoses in the siblings [23]. In multiplex families, subjects with ASD and the *MET* promoter risk variant have more severe social and communication symptoms [24]. Neuroimaging studies demonstrated that the same risk variant is associated with altered functional and structural connectivity in all scanned individuals but with more impacted phenotypes in subjects with ASD compared to typical controls [25]. Finally, the risk variant also is associated with distinct gray matter growth patterns in specific regions of the human brain [26].

Though recapitulating human brain-related clinical disorders precisely in rodents is not possible, behavioral analysis is very useful for translating human genetic and functional studies with developmental, cellular, and physiological changes in animal models in which there is genetically reduced or eliminated expression of ASD risk genes [27–29]. The present studies were designed to determine whether the *Met*-related cellular and electrophysiological phenotypes have specific behavioral consequences. Because constitutive *Met* null mice are embryonic lethal [30], we generated two conditional mouse lines, deleting *Met* from specific neuronal populations. The *Emx1*^*cre*^ driver line [31] was used to delete *Met* from neurons arising in the dorsal pallium and the *Nestin*^*cre*^ driver line [32] to delete *Met* from all neural cells. We report that the behavioral phenotypes vary by line and gene dose, consistent with a complex and heterogeneous impact of reducing *Met* expression in specific circuits, brain circuits in the mouse.

## Methods

### Mice

*Met*^*fx*/*fx*^/*Emx1*^*cre*^ animals were generated as previously described [11]. Briefly, conditional Met^fx/fx^/Emx1^cre^ mutant mice were produced by mating mice homozygous for a *Met* allele, in which exon 16 is flanked by loxP sites originally generated in the 129SV mouse strain [33] (courtesy of Dr. Snorri Thorgeirsson, NIH/Center for Cancer Research, Bethesda, MD), to *Emx1*^*cre*^ mice originally generated in the 129S2/SvPas strain [31] (courtesy of Dr. Kevin Jones, University of Colorado, Boulder, CO) that were also heterozygous for the floxed allele (*Met*^*fx*/*+*^/*Emx1*^*cre*^). Conditional *Met*^*fx*/*fx*^/*Nestin*^*cre*^ mutant mice were generated by mating mice homozygous for *Met* allele to Nestin^cre^ animals purchased from Jackson Laboratory (Strain Name: B6Cg-Tg(Nes-cre)1Kln/J) that were also heterozygous for the floxed allele (*Met*^*fx*/*+*^/*Nestin*^*cre*^). All breeding lines *Met*^*fx*/*fx*^, Met^fx/+^/Emx1^cre^, and *Met*^*fx*/*+*^/*Nestin*^*cre*^ were back-crossed onto the C57BL/6^J^ background (purchased from Jackson Laboratory) for greater than 10 generations, and their progeny (i.e., *Met*^*fx*/*fx*^/*Emx1*^*cre*^ (knockout (KO)), *Met*^*fx*/*+*^/*Emx1*^*cre*^ (heterozygous (Het)), *Met*^*fx*/*fx*^/*Nestin*^*cre*^ (KO), *Met*^*fx*/*+*^/*Nestin*^*cre*^ (Het), and littermate control (wildtype (WT)) mice) were genotyped via polymerase chain reaction (PCR) as previously described [11]. The PCR primer set for cre was forward 5′-TCGATG CAACGAGTGATGAG-3′ and reverse 5′-TTC GGC TAT ACG TAA CAG GG-3′ to produce a 481-bp PCR product.

Animals were housed on ventilated racks with their littermates (either WT and KO or WT, Het, and KO), with 12-h light-dark cycle (5:00 am–5:00 pm), and access to food and water ad libitum. Only adult male mice between postnatal day (P) 90 and P140 were used for behavioral analysis. All experiments conformed to the guidelines set forth by the University of Southern California Institutional Animal Care and Use Committee and the National Institutes of Health.

### Behavior

A battery of behavioral tests [28, 34] were used to assess cognitive, emotional, and social impairments that are observed in multiple neurodevelopmental disorders, including ASD, and that are, in part, subserved by circuits that express *Met* developmentally [10, 11]. Additional assays were performed to assess motor capacity. The sequence of tasks was such that simple motor tasks (rotarod and activity chamber) were performed first, followed by more complex testing of baseline cognition and anxiety (elevated plus maze, marble burying, spontaneous alternation). This was followed by sociability, social novelty preference and olfactory dishabituation, and lastly contextual and cued fear conditioning. Multiple cohorts of animals were run through the battery of tests. Within each cohort, WT animals were always pair housed either with KO animals or, for the *Met*^*fx*/*fx;fx*/*+*^/*Nestin*^*cre*^ cohorts, with KO and Het. Our behavioral battery assessment of the *Met*^*fx*/*fx*^/*Emx1*^*cre*^ cohorts revealed no significant differences between Het and WT offspring; so, our breeding schema was designed to favor production of KO animals. All animals in a cohort (WT, KO, Hets) were run simultaneously to control for potential environmental confounds. All behavioral assays were performed during the light portion of the circadian cycle between 6:30 am and 12:30 pm. Mice were habituated to transportation to the behavior rooms located in the vivarium and acclimated for a minimum of 30 min before each behavioral test. All tasks were performed by experimenters blind to the genotype of each animal.

## Data analysis

All behavioral coding and statistical analyses were performed by experimenters blind to the genotype of the animals. For tasks recorded by videotape (elevated plus maze, marble burying, T-maze, social novelty preference, and olfactory dishabituation), all behavioral codings were completed using a combination of CleverSys TopScan and Social Scan Software (CleverSys Inc., Reston, VA), and Mooses observation system [35]. For rotarod, activity chamber, contextual, and cued fear conditioning, behavioral codings were completed using the built-in software program from Med Associates for each task. Specific statistical analyses are described for each behavioral task. A *p* value less than 0.05 was considered significant.

### Specific behaviors

#### Rotarod

Performance on the rotarod was assessed to measure coordination and motor learning.

##### Apparatus

A Med Associates ENV-575M, Five Station Rota-Rod Treadmill USB for Mouse was used. The diameter of the rotating shaft was 3.2 cm; each lane was 5.7 cm wide, at an elevation of 16.5 cm.

##### Steady speed testing procedure

Mice were placed on the rotarod for 10 trials, 1 min each, under dim light (30 lx). The rotarod spins continuously at 32 RPM until the animal falls off the rotating shaft, thus breaking the s. The software calculates the latency to fall off the rotating shaft.

##### Accelerating testing procedure

Mice were placed on the rotarod for three trials, 5 min each, for three consecutive days, under dim light (30 lx). The rotarod accelerates from 4 to 40 RPM for each trial until the animal falls off the rotating shaft, thus breaking the photobeam. The software calculates the latency to fall off the rotating shaft.

##### Steady speed analysis

The amount of time the mouse spent on the rotarod per trial was calculated. A repeated measures ANOVA was used with genotype as a between subjects factor and trial as a within subjects factor. If the omnibus test detected a significant effect for genotype or genotype by behavior, a post hoc *t* test was performed to determine the trial in which the differences occurred.

##### Accelerating speed analysis

The amount of time the mouse spent on the rotarod was calculated for each trial. Averages for latency to fall were calculated for each of the 3 days. A repeated measures ANOVA was used with genotype as the between subjects factor and day as the within subjects factor. If the omnibus test detected a significant effect for genotype or genotype by behavior, a post hoc *t* test was performed to determine the day in which the differences occurred.

#### Activity chamber

Locomotor activity was assessed over a 30-min period in an activity chamber that was novel for each mouse.

##### Apparatus

Med Associates ENV-510 testing chambers within sound isolation cubicles were used. The chambers are 27 cm × 27 cm × 20.3 cm high with 16 infrared transmitters and receivers to detect movement in the *x*, *y*, and *z* planes. The house light and fan in the chambers remained on for the duration of the task.

##### Testing procedure

Mice were placed in the center of the chambers for free exploration over 30 min.

##### Analysis

The Med Associates Activity Monitor program detects infrared beam breaks, thus calculating total distance traveled in each plane (cm). Distance traveled during the 30-min trial, as well as data binned into serial 5-min time periods, was calculated for each animal. A repeated measures ANOVA was used with genotype as the between subjects factor and distance traveled as the within subjects factor. If the omnibus test detected a significant effect for genotype or genotype by behavior, a post hoc *t* test was performed to determine the time points at which differences occurred.

#### Elevated plus maze

To assess general anxiety, mice were tested on an elevated plus maze (EPM). Typically, mice spend greater time in the protected (closed) arms compared to the unprotected (open) arms.

##### Apparatus

A San Diego Instruments EPM for mice was used. The maze has a height of 38.74 cm, with each arm of the maze 5 cm wide, 30.5 cm long, and a 5 × 5-cm center compartment. The closed arms are protected by perimeter walls 15.24 cm high. The lighting intensity was set at 12 lx for the open arms, 1 lx for the closed arms, and 10 lx for the center.

##### Testing procedure

Mice were placed in the center of the maze, facing an open arm and provided 5 min to freely explore the maze.

##### Analysis

The number of entries into each closed and open arm, total arm entries, and the amount of time spent in the open and closed arms, and the center was calculated. Entries into the maze arms were defined as all four paws crossing into an arm from the center area. Entries and durations were automatically tabulated using the MazeScan suite of TopScan video analysis software (CleverSys Inc., Reston VA). Duration and entries were analyzed separately. A repeated measures ANOVA was performed for both measures with genotype as the between subjects factor and area of the maze (center or open/closed arm) as the within subjects factor. If the omnibus test detected a significant effect for genotype or genotype by behavior, a post hoc *t* test was performed to determine at which maze location the differences occurred.

#### Marble burying

Marble burying was performed to assess neophobia, general anxiety, and/or repetitive behaviors [36].

##### Apparatus

Allentown microVent wide rat cages, 39 cm long × 28.5 cm wide × 19 cm high, were used.

##### Testing procedure

Animals were habituated to a novel cage with 4.5 cm of Sani-Chip bedding (Absorption Corp., Ferndale, WA) for 30 min. After habituation, animals were briefly removed and 20 marbles were systematically arranged in the same test cage. Animals were then placed back into the cage and given 30 min to freely explore. The task was performed in a room with 30 lx lighting.

##### Analysis

The number of marbles that was buried (minimum 50 % coverage of the marble) at the end of the 30-min session was counted. A one-way ANOVA (genotype as between factor) was performed to determine statistical significance between genotypes.

#### Spontaneous alternation in T-maze

Spontaneous alternation in the T-maze was used to assess working memory and attention, although performance is known to be influenced by states of anxiety, arousal, and altered novelty preference [37, 38].

##### Apparatus

A San Diego Instruments T-maze for mice was used. Three enclosed arms comprise the maze, two of which are 15.24 cm in length each, and one arm 19.05 cm in length. Each arm is 5.08 cm wide, and center area is 5.08 cm by 5.08 cm. Wall height is 11.58 cm. The arms of the maze were illuminated between 12 and 18 lx during testing.

##### Testing procedure

Mice were placed in the center of the maze facing one arm and given 8 min to explore the maze. Each session was recorded by a video camera positioned above the maze. An arm visit was counted when all four paws were moved into an arm.

##### Analysis

A triple alternation in the T-maze was defined as a visit to each of the three arms sequentially. Same arm returns (SAR) indicated that an animal returned to the same arm it had exited. Alternate arm returns (AAR) was defined as an alternation between two arms. The percent spontaneous alternation (%SA) was calculated by dividing the number of triple alternations by the number of possible alternations [# alternations/(# total arm entries−2) × 100], as described previously [39–45]. Chance performance is based on three arms with 3^3^ possible combination of entries, but only six of those combinations result in a triplet; thus chance is equal to (6/27)*100 %, or 22.2 %. Percent same arm and alternate arm returns (% SAR and % AAR) were calculated as a ratio of returns to total number of entries multiplied by 100 (SAR or AAR/total entries) *100). A one-way ANOVA (genotype as the between subjects factor) was used to determine statistical significance for total arm entries, % SA, % AAR, and % SAR.

#### Olfactory dishabituation

Olfactory detection, habituation, and dishabituation to social and non-social odorants were measured in all mice. This specific task takes advantage of a rodent’s ability to rapidly habituate to an odor, by adapting a task that has been utilized to demonstrate deficits in this modality in mutant mice [46–48]. This task is divided into five odor presentation blocks in the following order: water (baseline), non-social #1, non-social #2, social #1, and social #2. During each block, mice typically show high levels of sniffing during the first presentation of the odor, but rapidly habituate, reflected in decreased sniffing time across the second and third presentations of the same odor. When a new odor is presented, sniffing time increases (dishabituation), indicating that the mouse can distinguish between the two odors.

##### Apparatus

An Allentown microVent mouse cage measuring 29.5 cm long by 18.5 cm wide by 13 cm high was used. The empty wire food rack was placed on top of the cage to hold the cotton swab during testing.

##### Testing procedure

The mouse was placed in novel cage for a 30-min acclimation, followed by three 2-min presentations of (1) 150-μL water, (2) 150-μL lemon extract, (3) 150-μL vanilla extract, (4) a cotton swab swabbed on the bottom of stranger mouse #1’s homecage, and (5) a cotton swab swabbed on the bottom of stranger mouse #2’s homecage. Pure lemon and vanilla extract (McCormick and Company, Inc.) were applied at full concentration. The odor presentation was counterbalanced so half the animals received lemon extract presentations first, followed by vanilla, and the other half of the animals had the reverse presentation. The task was performed in a room with standard lighting (65–70 lx) to allow for proper side view videotaping of the behavioral task.

##### Analysis

The time spent sniffing the cotton swab was recorded for each odor presentation. A repeated measures ANOVA was performed with genotype as the between subjects factor, and odorant presented as the within subjects factor. If the omnibus test detected a significant effect for genotype or genotype by behavior, a post hoc *t* test was performed to determine for which odorant sniffing behavior differences occurred.

#### Social novelty preference

Social behavior was assayed using a modified three-chamber arena task. This task probes general sociability and preference for social novelty, using a well-described paradigm [28, 49] in a custom-designed three-chamber arena.

##### Apparatus

The equipment used for this task was a custom designed Plexiglas three-chamber arena 63 cm long by 42 cm wide by 23 cm high. The two outer chambers are 24.5 cm long × 42 cm wide × 23 cm high, and the inner compartment between the two outer chambers is 11.5 cm long × 42 cm wide × 23 cm high. The inner compartment has two walls with small entryways (10 cm high by 10 cm long, 14.5 cm from end of chamber) to allow free exploration of all three chambers when doors are in the open position. The doors measure 10.5 cm × 28 cm and remain open for the duration of the experiment.

##### Testing procedure

The experimental mouse was placed in the central compartment and given 10 min to explore the arena with an empty inverted wire cup (Spectrum Diversified Designs, Streetsboro, OH, USA) present in the left and right compartments. A stimulus mouse was then placed inside an inverted wire cup on one side, and the experimental mouse was allowed to explore the arena for an additional 10 min (sociability phase). A second novel stimulus mouse was then placed under an inverted wire cup on the opposite side, and the experimental mouse was allowed to explore the arena for another 10 min (social novelty preference). The task was performed in a room with 30 lx lighting.

##### Analysis

The time spent in each of the three chambers and time spent investigating each of the wire cups were calculated for both phases of the test (sociability and social novelty preference). A two-way ANOVA was performed with genotype as the between subjects factor and either chamber time or time spent investigating cup as the within subjects factor during both sociability and social novelty preference trials. If the omnibus test detected a significant effect for genotype or genotype by behavior, a post hoc *t* test was performed to determine at which maze location the differences occurred.

#### Fear conditioning

Learning and memory for both contextual and cue-specific fear conditioning was assayed. Cohorts of WT, *Met*^*fx*/*fx*^/*Emx1*^*cre*^, *Met*^*fx*/*fx*^/Nestin^cre^, and *Met*^*fx*/*+*^/Nestin^cre^ mice were tested in contextual fear conditioning. *Met*^*fx*/*fx*^/Nestin^cre^ and *Met*^*fx*/*+*^/Nestin^cre^ mice animals were also tested in the cued fear conditioning paradigm based on initial results in the contextual fear conditioning paradigm. Individual animals were tested in either the contextual or the cued fear conditioning paradigm; no animal was tested in both paradigms to avoid confounds due to the influence of one task on subsequent fear testing.

##### Apparatus

Med Associates MED-VFC-NIR-M, NIR Video Fear Conditioning Systems for Mouse were used. Fear conditioning chambers were 30 × 25 × 25 cm housed within sound attenuating chambers.

##### Testing procedure

Contextual fear conditioning:

Training: Animals were placed into the dark sound-attenuating cubicle and acclimated for 3 min. Animals were then presented with five 2-s 0.5-mA footshocks separated by 220-s intertrial intervals. Two minutes after the last shock, mice were placed into their homecage for 24 h.

Test: The next day, mice were placed into the same dark sound-attenuating cubicle. Animals were given 8 min to explore without footshocks presented.

Cued (auditory) fear conditioning

Habituation in context A: Animals were placed into the dark sound-attenuating cubicle and acclimated for 30 min, followed by return to their homecage for 24 h.

Training in context A: Animals were placed into the dark sound-attenuating cubicle and given 3 min to acclimate. Animals were then presented with five 2-s 0.3-mA footshocks that were preceded by a 30-s 85-dB 5-kHz tone that terminated simultaneously with the termination of the footshock. Footshocks were separated by 180-s intertrial intervals. Three minutes after the last shock, mice were returned to their homecage for 24 h.

Contextual test in context A: Animals were placed into the dark sound-attenuating cubicle for 8 min and then returned to their homecage for 20 min before the cue test.

Cue test in context B: Animals were placed into the dark sound-attenuating cubicle with new floor and wall inserts to change the appearance of the cubicle and given 3 min to acclimate. Animals were then presented with 10 presentations of the 30-s 85-dB 5-kHz tone separated by 60-s intertrial intervals. One minute after the last tone, animals were returned to their homecage for 24 h.

Extinction tests in context B: Animals were placed into the dark sound-attenuating cubicle with new floor and wall inserts to change the appearance of the cubicle and given 3 min to acclimate. Animals were then presented with 10 presentations of the 30-s 85-dB 5-kHz tone separated by 60-s intertrial intervals. One minute after the last tone, animals were returned to their homecage.

##### Analysis

Video freeze by Med Associates was used to calculate percent time spent freezing during learning and memory trials of contextual and cued fear conditioning. A repeated measures ANOVA was performed with genotype as the between subjects factor and freezing during trials as the within subjects factor. If the omnibus test detected a significant effect for genotype or genotype by behavior, a post hoc *t* test was performed to determine during which trials the differences occurred.

## Results and discussion

Multiple cohorts of animals were run within the same animal vivarium. Within each cohort, there were always matched WT and KO (and Het for Nestin^cre^) run simultaneously to control for possible environmental confounds. All cohorts were collapsed into a single dataset for each driver line (Emx1^cre^ or Nestin^cre^) as there were no statistically significant differences in patterns of data generated between cohorts. Data are presented separately for the *Met*^*fx*/*fx*^/*Emx1*^*cre*^ line and the *Met*^*fx*/*fx;fx*/*+*^/Nestin^cre^ line.

### Met^fx/fx^/Emx1^cre^

Animals that were homozygous null for *Met* in structures that are derived from the dorsal pallium (including excitatory projection neurons in neocortex, olfactory bulb, CA1 neurons of the hippocampus, and cortical amygdala region) were tested in the battery of behavioral tests described above.

#### Steady speed rotarod

**Fig. 1:**
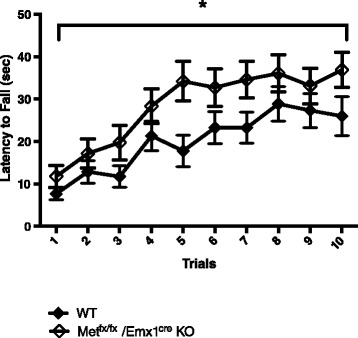
Performance on the steady speed rotarod. *Met* ^*fx*/*fx*^/*Emx1* ^*cre*^ KO animals demonstrated consistently better performance on the rotarod for all trials compared to their WT littermates (*p < 0.05)

*Met*^*fx*/*fx*^/*Emx1*^*cre*^ KO animals demonstrated better performance on the rotarod compared to their WT littermates, *F* (1, 52) = 4.245, *p* < 0.05 (Fig. 1). Both groups showed consistent improved performance across the 10 trials, *F* (9, 468) = 18.990, *p* < 0.0001. There was no interaction effect between genotype and trials, *F* (9, 468) = 1.063, *p* > 0.05.

#### Accelerating rotarod

A two-way repeated measures ANOVA revealed no significant differences between genotypes for motor learning on the accelerating rotarod, *p* > 0.05 (Table 1). Both groups showed consistent improved performance across the 3 days, *p* < 0.0001. There was no interaction effect between genotype and days, *p* > 0.05.

**Table 1:** Behavioral characterization of WT and Met^fx/fx^/Emx1^cre^ KO mice

	WT	Met^fx/fx^/Emx1^cre^ KO	*F* or *t* Statistic	*p* value
Activity chamber				
			Genotype, *F* (1, 51) = 12.26	*p* < 0.05
Total distance traveled(cm)	3317.67 ± 218.98	2339.74 ± 175.11	Time, *F* (5, 255) = 4.308	*p* < 0.001
			Interaction, *F* (5, 255) = 3.184	*p* < 0.01
Steady speed rotarod				
			Genotype, *F* (1, 52) = 4.249	*p* < 0.05
Average latency to fall(sec)	20.0185 ± 2.58	28.481 ± 3.19	Trial, *F* (9, 468) = 18.99	*p* < 0.0001
			Interaction, *F* (9, 468) = 1.063	*p* > 0.05
Accelerating rotarod				
			Genotype, *F* (1, 12) = 0.017	*p* > 0.05
Average latency to fall(sec)	151.43 ± 19.82	148.10 ± 16.14	Trial, *F* (2, 24) = 15.35	*p* < 0.0001
			Interaction, *F* (2, 24) = 0.024	*p* > 0.05
T-maze				
% SA	54.701 ± 1.679	47.4956 ± 2.401	*t*_51_ = 2.475	*p* < 0.05
% AAR	35.063 ± 1.554	38.576 ± 1.599	*t*_51_ = −1.576	*p* > 0.05
% SAR	3.891 ± 0.666	5.301 ± 0.907	*t*_51_ = −1.410	*p* > 0.05
#arm entries	35.852 ± 2.456	29.154 ± 1.859	*t*_51_ = 2.162	*p* < 0.05
Marble burying (#)	7.100 ± 1.567	9.200 ± 1.756	*t*_18_ = 0.8923	*p* > 0.05
EPM entries (#)				
			Genotype, *F* (1, 32) = 0.2539	*p* > 0.05
Closed arms	9.188 ± 1.697	8.882 ± 1.364	Arm entry, *F* (1, 32) = 65.93	*p* < 0.05
Open arms	1.706 ± 0.803	0.5882 ± 0.298	Interaction, *F* (1, 32) = 0.2081	*p* > 0.05
EPM arm duration (%)				
			Genotype, *F* (1, 32) = 0.3463	*p* > 0.05
Closed arms	88.882 ± 3.169	86.863 ± 5.017	Arm duration, *F* (1, 32) = 220.8	*p* < 0.0001
Open arms	4.3137 ± 1.505	3.000 ± 1.649	Interaction, *F* (1, 32) = 0.2508	*p* > 0.05
Sociability (sec)				
			Genotype, *F* (1, 52) = 1.463	*p* > 0.05
Side with mouse	312.897 ± 14.864	317.225 ± 11.721	Chamber side, *F* (1, 52) = 74.25	*p* < 0.05
Side with empty cup	185.271 ± 12.428	162.44 ± 11.885	Interaction, *F* (1, 52) = 0.6864	*p* > 0.05
Social novelty (sec)				
			Genotype, *F* (1, 52) = 0.001	*p* > 0.05
Familiar mouse side	227.609 ± 13.819	235.362 ± 17.602	Chamber side, *F* (1, 52) = 1.545	*p* > 0.05
Novel mouse side	259.723 ± 14.579	252.667 ± 14.533	Interaction, *F* (1, 52) = 0.1387	*p* > 0.05
Sociability (sec)				
			Genotype, *F* (1, 52) = 0.005652	*p* > 0.05
Cup with mouse	62.795 ± 4.653	60.152 ± 5.095	Cup, *F* (1, 52) = 214.6	*p* < 0.05
Empty cup	11.818 ± 1.606	12.645 ± 1.254	Interaction, *F* (1, 52) = 0.2665	*p* > 0.05
Social novelty (sec)				
			Genotype, *F* (1, 52) = 0.02144	*p* > 0.05
Cup with familiar mouse	23.087 ± 3.234	21.624 ± 2.801	Cup, *F* (1, 52) = 63.83	*p* < 0.05
Cup with novel mouse	46.756 ± 5.329	46.817 ± 4.221	Interaction, *F* (1, 52) = 0.06198	*p* > 0.05
Contextual fear (%)				
Baseline	0.04976 ± 0.0497	0.3007 ± 0.1955	Genotype, *F* (1, 31) = 0.6584	*p* > 0.05
Training	28.73 ± 3.2649	32.72 ± 2.6444	Trial, *F* (2, 62) = 110.5	*p* < 0.0001
Test	29.04 ± 3.2954	30.81 ± 3.4346	Interaction, *F* (2, 62) = 0.3224	*p* > 0.05

#### Activity chamber

**Fig. 2:**
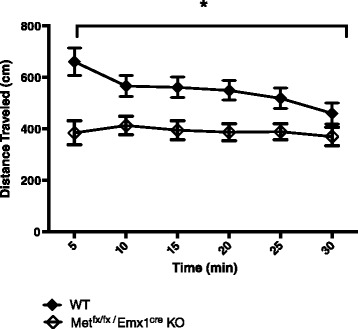
Locomotion in the activity chamber. Compared to their WT littermates, *Met* ^*fx*/*fx*^/*Emx1* ^*cre*^ KO animals were hypoactive in the activity chamber (*p < 0.005)

*Met*^*fx*/*fx*^/*Emx1*^*cre*^ KO animals were hypoactive compared to their WT littermates, with a statistically significant main effect for genotype, *F* (1, 51) = 12.260, *p* < 0.005 (Fig. 2). There was a main effect for time, *F* (5, 255) = 4.308, *p* < 0.001, with all animals, independent of genotype, displaying more exploratory behavior at the beginning of the 30-min trial, followed by reduced exploration. Additionally, there was an interaction effect, *F* (5, 255) = 3.184, *p* < 0.01, with the WT animals exhibiting more exploratory behavior during the first 5 min, then tapering off. In contrast, the KO animals showed a more consistent activity level across the 30-min task.

#### Elevated plus maze

*Met*^*fx*/*fx*^/*Emx1*^*cre*^ KO animals did not exhibit anxiety-like behavior in the EPM task (Table 1). Separate two-way ANOVAs were performed for duration and arm entries in the EPM. For duration, there was no main effect for genotype, *p* > 0.05. There was a main effect for location in the maze, with animals of both genotypes spending more time in the closed arms, *p* < 0.0001. Furthermore, there was no interaction between genotype and arm location, *p* > 0.05. Similarly, analysis of arm entries revealed there was no main effect for genotype, *p* > 0.05. There was a main effect for location in the maze, with all animals spending more time in the closed arms than open arms, *p* < 0.05. Furthermore, there was no interaction between genotype and location in the maze, *p* > 0.05.

#### Marble burying

There was no anxiety-like nor repetitive behaviors exhibited by *Met*^*fx*/*fx*^/*Emx1*^*cre*^ KO animals in this task (Table 1). A *t* test revealed no significant difference between genotypes for the total number of marbles buried at the end of the 30-min trial, *p* > 0.05.

#### T-maze

**Fig. 3:**
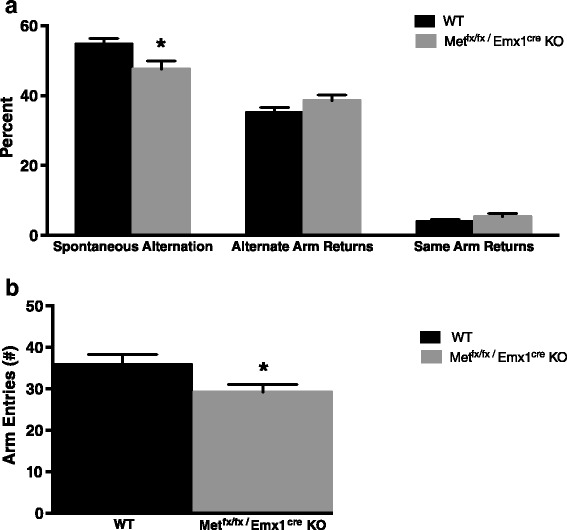
Spontaneous alternation in the T-maze. **a** *Met* ^*fx*/*fx*^/*Emx1* ^*cre*^ KO animals showed significant impairment in percent spontaneous alternation, while alternate arm returns and same arm returns were equivalent between the two genotypes. **b** *Met* ^*fx*/*fx*^/*Emx1* ^*cre*^ KO animals also displayed reduced exploration in the T-maze compared to WT animals (*p < 0.05)

*Met*^*fx*/*fx*^/*Emx1*^*cre*^ KO animals showed significant impairment in spontaneous alternation, *t*_51_ = 2.475, *p* < 0.05 (Fig. 3a). However, neither alternate arm returns nor same arm returns were significantly different between groups, *t*_51_ = −1.576, *p* > 0.05, and *t*_51_ = −1.259, *p* > 0.05, respectively. Consistent with their hypoactivity in the activity chamber, KO animals also displayed reduced exploration in the T-maze, with significantly diminished total arm entries, *t*_51_ = 2.162, *p* < 0.05 (Fig. 3b). Because spontaneous alternation, alternate arm, and same arm returns were calculated by including total arm entries as a moderator, the decreased locomotor activity in the T-maze was not a confounder of the spontaneous alternation findings.

#### Sociability and social novelty preference

Overall, there were no differences in either sociability scores or in social novelty preference scores between *Met*^*fx*/*fx*^/*Emx1*^*cre*^ KO and WT animals (Table 1). Time in each chamber and time sniffing specific cups were analyzed separately. For chamber time, during the sociability trial, there was no main effect for genotype, *p* > 0.05. There was a main effect for chamber location during sociability *p* < 0.05, with all animals spending more time in the chamber with the stimulus mouse. Furthermore, there was no interaction effect, *p* > 0.05. For chamber time, during the *social novelty preference* trial, there was no main effect for genotype, *p* > 0.05. Interestingly, there also was no main effect for chamber location, *p* > 0.05. Furthermore, there was no interaction effect, *p* > 0.05. In comparing cup-sniffing times, there also were no differences either in sociability or in social novelty preference scores between the two groups. Both groups of animals spent more time sniffing the cup with the stimulus mouse, *p* < 0.05, and there was no main effect for genotype during the sociability trial, *p* > 0.05. Furthermore, there was no interaction between genotype and cup sniffing, *p* > 0.05. During the social novelty preference trial, both groups of animals spent more time sniffing the cup with the novel mouse, *p* < 0.05, but there was no main effect for genotype, *p* > 0.05. Additionally, there was no interaction between genotype and cup sniffing, *p* > 0.05.

#### Olfactory dishabituation

A two-way repeated measures ANOVA revealed there were no significant differences between genotypes for sniffing behaviors during the olfactory test, *p* > 0.05 (Table 1).There was a significant main effect for trial, *p* < 0.001, with the social trials eliciting more sniffing time. Furthermore, there was no interaction effect, *p* > 0.05, with both groups of animals spending similar times sniffing odorants.

#### Contextual fear conditioning

A repeated measures ANOVA for fear conditioning revealed no significant differences between genotypes, *p* > 0.05 (Table 1). There was a main effect for fear conditioning trial, *p* < 0.0001, with all animals displaying significantly increased freezing during the training and retention test compared to baseline. Furthermore, there was no interaction between genotype and trial, *p* > 0.05.

### Met^fx/fx;fx/+^/Nestin^cre^

Animals that were either heterozygous (*Met*^*fx*/*+*^) or homozygous (*Met*^*fx*/*fx*^) null for *Met* in peripheral and central neural structures were tested in the behavioral battery described above.

#### Steady speed rotarod

In contrast to the *Met*^*fx*/*fx*^/*Emx1*^*cre*^ KO mice, a two-way repeated measures ANOVA revealed no significant differences between any of the *Nestin*^*cre*^-derived genotypes for performance on the rotarod, *p* > 0.05 (Table 2). All animals showed improved performance across trials, *p* < 0.0001. Furthermore, there was no interaction effect between genotype and rotarod trials, *p* > 0.05.

**Table 2:** Behavioral characterization of WT, Met^fx/+^/Nestin^cre^ Het, and Met^fx/fx^/Nestin^cre^ KO mice

	WT	Met^fx/+^/Nestin^cre^ Het	Met^fx/fx^/Nestin^cre^ KO	*F* Statistic	*p* value
Activity chamber					
				Genotype, *F* (2, 55) = 2.491	*p* > 0.05
Total distance traveled(cm)	2807.3 ± 197.9	3445.5 ± 236.0	2842.9 ± 140.5	Time, *F* (5, 275) = 18.09	*p* < 0.0001
				Interaction, *F* (5, 275) = 1.410	*p* > 0.05
Steady speed rotarod					
				Genotype, *F* (2, 55) = 1.971	*p* > 0.05
Latency to fall(sec)	27.417 ± 2.635	14.271 ± 2.016	20.033 ± 3.363	Trial, *F* (9, 495) = 9.686	*p* < 0.0001
				Interaction, *F* (9, 495) = 1.002	*p* > 0.05
Accelerating rotarod					
				Genotype, *F* (2, 17) = 0.033	*p* > 0.05
Latency to fall(sec)	150.39 ± 15.80	155.49 ± 14.76	148.38 ± 14.71	Trial, *F* (2, 34) = 18.50	*p* < 0.0001
				Interaction, *F* (2, 34) = 1.737	*p* > 0.05
T-maze					
% SA	44.837 ± 2.456	49.408 ± 2.568	50.113 ± 2.181	*F* (2, 53) = 1.417	*p* > 0.05
% AAR	36.794 ± 2.114	31.852 ± 1.356	31.123 ± 2.096	*F* (2, 53) = 2.408	*p* > 0.05
% SAR	7.354 ± 1.1011	7.840 ± 1.198	8.192 ± 1.170	*F* (2, 53) = 0.1350	*p* > 0.05
#arm entries	27.963 ± 1.778	30.429 ± 2.467	33.333 ± 2.131	*F* (2, 53) = 1.742	*p* > 0.05
Marble burying (#)	8 ± 1.076	10 ± 1.742	7.0 ± 1.366	*F* (2, 53) = 0.6750	*p* > 0.05
EPM entries (#)					
				Genotype, *F* (2, 54) = 1.490	*p* > 0.05
Closed arms	9.5 ± 0.946	11.21 ± 1.085	9.8 ± 1.147	Arm entries, *F* (1, 54) = 147.8	*p* < 0.0001
Open arms	1.75 ± 0.413	3.21 ± 0.594	2.4 ± 0.523	Interaction, *F* (2, 54) = 0.06371	*p* > 0.05
EPM duration (%)					
				Genotype, *F* (2, 54) = 0.0	*p* > 0.05
Closed arms	81.037 ± 2.725	63.268 ± 5.701	75.057 ± 3.494	Arm duration, *F* (2, 108) = 228.1	*p* < 0.0001
Open arms	8.102 ± 2.276	13.523 ± 5.628	8.736 ± 2.077	Interaction, *F* (2, 108) = 4.299	*p* < 0.005
Sociability (sec)					
				Genotype, *F* (2, 53) = 1.032	*p* > 0.05
Side with mouse	335.257 ± 12.743	340.71 ± 11.666	310.944 ± 13.768	Chamber side, *F* (2, 106) = 197.6	*p* < 0.0001
Side with empty cup	165.806 ± 10.710	144.05 ± 10.953	199.303 ± 14.887	Interaction, *F* (2, 106) = 2.493	*p* < 0.05
Social novelty (sec)					
				Genotype, *F* (2, 53) = 0.4669	*p* > 0.05
Familiar mouse side	239.276 ± 15.473	275.184 ± 20.999	239.899 ± 24.126	Chamber side, *F* (2, 106) = 47.53	*p* < 0.0001
Novel mouse side	256.915 ± 14.165	213.617 ± 17.351	256.977 ± 24.069	Interaction, *F* (2, 106) = 1.174	*p* > 0.05
Sociability (sec)					
				Genotype, *F* (2, 53) = 0.7821	*p* > 0.05
Cup with mouse	76.390 ± 5.722	72.092 ± 5.533	64.901 ± 5.795	Cup time, *F* (1, 53) = 212.2	*p* < 0.0001
Empty cup	13.486 ± 1.589	12.605 ± 2.816	14.391 ± 2.338	Interaction, *F* (2, 53) = 0.9274	*p* > 0.05
Social novelty (sec)					
				Genotype, *F* (2, 53) = 0.2960	*p* > 0.05
Cup with familiar mouse	25.686 ± 3.224	27.324 ± 3.362	25.807 ± 3.938	Cup time, *F* (1, 53) = 23.81	*p* < 0.0001
Cup with novel Mouse	46.11 ± 3.858	38.358 ± 4.95	41.836 ± 4.558	Interaction, *F* (2, 53) = 0.7666	*p* > 0.05
Cue fear training (%)					
				Genotype, *F* (2, 21) = 1.206	*p* > 0.05
Average freezing	27.681 ± 3.621	20.959 ± 9.090	24.956 ± 2.421	Trial, *F* (4, 84) = 31.76	*p* < 0.0001
				Interaction, *F* (4, 84) = 0.8341	*p* > 0.05
Cue fear context test (%)					
Average freezing	25.501 ± 2.778	21.132 ± 6.485	24.408 ± 4.978	*F* (2, 21) = 0.201	*p* > 0.05
Cue fear cue test (%)					
				Genotype, *F* (2, 21) = 3.156	*p* > 0.05
Average freezing	61.120 ± 1.062	72.072 ± 2.478	78.351 ± 2.758	Trial, *F* (9, 189) = 1.398	*p* > 0.05
				Interaction, *F* (9, 189) = 0.9551	*p* > 0.05
Cue fear extinction 1 (%)					
				Genotype, *F* (2, 21) = 1.032	*p* > 0.05
Average freezing	47.999 ± 1.515	60.181 ± 2.219	56.860 ± 2.118	Trial, *F* (9, 189) = 1.975	*p* < 0.05
				Interaction, *F* (9, 189) = 0.6945	*p* > 0.05
Extinction 2					
				Genotype, F (2, 21) = 0.9524	*p* > 0.05
Average freezing	34.572 ± 1.967	40.725 ± 3.196	45.103 ± 1.575	Trial, *F* (9, 189) = 1.480	*p* > 0.05
				Interaction, *F* (9, 189) = 1.196	*p* > 0.05
Extinction 3					
				Genotype, *F* (2, 21) = 0.2070	*p* > 0.05
Average freezing	30.900 ± 2.513	29.381 ± 1.754	36.167 ± 1.686	Trial, *F* (9, 189) = 1.026	*p* > 0.05
				Interaction, *F* (9, 189) = 1.180	*p* > 0.05

#### Accelerating rotarod

A two-way repeated measures ANOVA revealed no significant differences between genotypes for motor learning on the accelerating rotarod, *p* > 0.05 (Table 2). All animals showed consistent improved performance across the 3 days, *p* < 0.0001. Furthermore, there was no interaction effect between genotype and days, *p* > 0.05.

#### Activity chamber

Whereas the *Met*^*fx*/*fx*^/*Emx1*^*cre*^ KO mice exhibited activity differences compared to WT, a two-way repeated measures ANOVA revealed no significant differences between any of the *Nestin*^*cre*^-derived genotypes, *p* > 0.05, indicating that there was no difference in total distance traveled by all animals (Table 2). Additionally, all animals, independent of genotype, displayed more exploratory behavior at the beginning of the 30-min trial and then reduced their exploration, with a main effect for time *p* < 0.0001. Furthermore, there was no interaction effect between genotype and time in activity chamber, *p* > 0.05.

#### Elevated plus maze

**Fig. 4:**
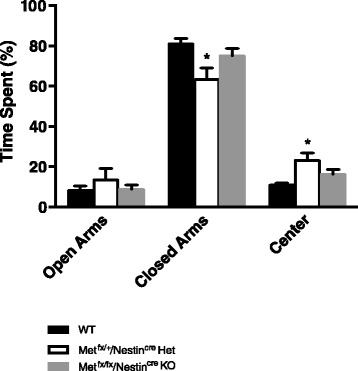
Elevated plus maze performance. *Met* ^*fx*/*+*^/*Nestin* ^*cre*^ Het mice spent significantly more time in the center and less time in the closed arms of the elevated plus maze as compared to WT animals. (*p < 0.05). There were no differences in time spent in the open arms between groups

Separate two-way repeated measures ANOVAs were performed for both duration and entries in the EPM (Table 2). For duration, there was no main effect for genotype, *p* > 0.05. There was a main effect for location in the maze, all animals spent more time in the closed arms, *p* < 0.0001. In contrast to the findings with the *Met*^*fx*/*fx*^/*Emx1*^*cre*^ KO mice, there was a significant interaction between genotype and arm location, *p* < 0.005. A Tukey’s post hoc analysis revealed *Met*^*fx*/*+*^/*Nestin*^*cre*^ Het was significantly different from WT in both the closed arm and center time, with the *Met*^*fx*/*+*^/*Nestin*^*cre*^ Het mice spending more time in the center portion of the arena (Fig. 4). Analysis of arm entries revealed there was no main effect for genotype, *p* > 0.05. There was a main effect for location in the maze; all animals returned to the closed arms more often than open arms, *p* < 0.0001. Furthermore, there was no interaction, *p* > 0.05.

#### Marble burying

A one-way ANOVA revealed no significant differences in total number of marbles buried between genotypes, *p* > 0.05 (Table 2).

#### T-maze

Distinct from the *Met*^*fx*/*fx*^/*Emx1*^*cre*^ KO mice, a one-way ANOVA found no differences in spontaneous alternation for any of the *Nestin*^*cre*^-derived genotypes, *p* > 0.05 (Table 2). Neither alternate arm returns, same arm returns, nor total arm entries were significantly different between groups, *p* > 0.05.

#### Sociability and social novelty preference

**Fig. 5:**
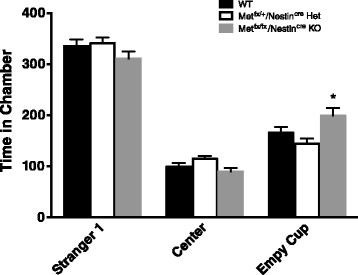
Sociability in the three-chamber task. While all animals demonstrated sociability (more time in chamber with stranger mouse than empty cup), *Met* ^*fx*/*fx*^/*Nestin* ^*cre*^ KO mice spent significantly more time in the chamber with the empty wire cup compared to *Met* ^*fx*/*+*^/*Nestin* ^*cre*^ Het animals. (*p < 0.05). There were no significant differences in center time or in the time spent in the chamber with the stranger mouse

Two-way repeated measures ANOVA was conducted for sociability and social novelty preference scores using both chamber time and cup sniffing times for separate analyses (Table 2). For the chamber time during the sociability trial, there was no main effect for genotype, *p* > 0.05. There was a main effect for chamber location during sociability, *p* < 0.0001, with all animals spending more time in the chamber with the stimulus mouse. Interestingly, there was an interaction effect, *p* < 0.05. A Tukey’s post hoc analysis revealed *Met*^*fx*/*fx*^/*Nestin*^*cre*^ KO animals spent significantly more time in the chamber with an empty wire cup than the *Met*^*fx*/*+*^/*Nestin*^*cre*^ Het animals (Fig. 5). For chamber time during the social novelty preference trial, there was no main effect for genotype, *p* > 0.05. There was a main effect for chamber location during the test with all animals spending more time on the side of the chamber where the novel mouse was located, *p* < 0.0001. Furthermore, there was no interaction effect, *p* > 0.05. When comparing cup-sniffing times, there were no differences either in sociability or in social novelty preference scores between the groups. During the sociability trial, there was no main effect for genotype, *p* > 0.05. Both groups of animals spent more time sniffing the cup with the stimulus mouse, *p* < 0.0001. Furthermore, there was no interaction between genotype and cup sniffing, *p* > 0.05. During the social novelty preference trial, there was no main effect for genotype, *p* > 0.05. All animals spent more time sniffing the novel mouse, *p* < 0.0001. Additionally, there was no interaction between genotype and cup sniffing, *p* > 0.05.

#### Olfactory dishabituation

There were no significant differences between genotypes during olfactory dishabituation, *p* > 0.05 (Table 2). There was a significant main effect for trial, *p* < 0.0001, with the social trials eliciting more sniffing time. Furthermore, there was no interaction effect between genotype and sniffing behavior, *p* > 0.05, with all animals showing similar sniffing behaviors across odorants.

#### Contextual fear conditioning

**Fig. 6:**
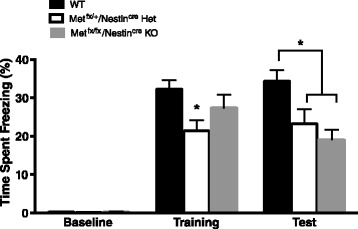
Contextual fear learning and memory. *Met* ^*fx*/*+*^/*Nestin* ^*cre*^ Het mice exhibited significantly impaired freezing during contextual fear training compared to WT animals. Both the *Met* ^*fx*/*+*^/*Nestin* ^*cre*^ Het and *Met* ^*fx*/*fx*^/*Nestin* ^*cre*^ KO animals also showed significantly blunted freezing during the retention test compared to WT animals (*p < 0.05)

In contrast to the *Met*^*fx*/*fx*^/*Emx1*^*cre*^ KO mice, there were significant differences between genotypes for contextual fear conditioning, *F* (2, 53) = 5.748, *p* < 0.01 (Fig. 6). Additionally, there was a main effect for fear conditioning phase, *F* (2, 106) = 152.3, *p* < 0.0001, with animals displaying significantly increased freezing during the training session and retention test compared to baseline. Furthermore, there was also a significant interaction effect, *F* (2, 106) = 5.209, *p* < 0.001. Tukey’s multiple comparisons test revealed *Met*^*fx*/*+*^/*Nestin*^*cre*^ Het mice exhibited significantly impaired freezing during contextual fear training compared to WT (*p* < 0.05), while *Met*^*fx*/*fx*^/*Nestin*^*cre*^ KO animals display a non-significant trend toward reduced freezing. Additionally, Tukey’s multiple comparison test also revealed that both the *Met*^*fx*/*+*^/*Nestin*^*cre*^ Het and *Met*^*fx*/*fx*^/*Nestin*^*cre*^ KO animals showed significantly blunted freezing during the retention test compared to WT, *p* < 0.05.

#### Cued (auditory) fear conditioning

Training, testing, and extinction trials were analyzed separately with two-way repeated measures ANOVAs to determine differences in rates of learning across genotypes (Table 2). For the acquisition of cued fear conditioning, there was no main effect for genotype, *p* > 0.05. There was a main effect for trial, *p* < 0.0001, with all animals showing increased freezing times to the presentation of the cue with more footshock exposures. There was no interaction effect between genotype and trials, *p* > 0.05. The following day, there was very little freezing during the context test. There were no differences in the context test between genotypes, *p* > 0.05. During the cue test, however, animals showed significant freezing times. There was no main effect for genotype, *p* > 0.05. There also was no main effect for trial, *p* > 0.05, with all animals showing consistent freezing across trials. Furthermore, there was no genotype by trial interaction, *p* > 0.05. During all three extinction tests, there was no main effect for genotype, *p* > 0.05. Only during the first extinction test was there a main effect for trial, *p* < 0.05, with all animals showing decreased freezing across trials. The remaining two extinctions showed no main effect for trial, *p* > 0.05. Furthermore, for all three extinction tests, there was no genotype by trial interaction, *p* > 0.05.

## Conclusions

Here, we report that genetic disruption of *Met* expression results in distinct phenotypes depending upon the neuronal populations that are targeted. The findings are not surprising, given that multiple studies across species have shown that disruption of MET signaling genetically results in distinct cellular phenotypes, depending upon the central or peripheral neural structure in which gene expression is manipulated [3, 5, 8, 15, 30, 50]. For example, our morphological and electrophysiological studies revealed differences in synapse development that alter excitatory drive onto deep layer pyramidal neurons from input located in superficial layers in the neocortex and early excitatory synapse maturation in the hippocampus. It is important to emphasize that specific altered phenotypes are evident in only select subpopulations of neurons. For example, increased excitatory drive is expressed by a subset of layer V cortical-striatal neurons, but not cortico-pontine neurons [15]. Moreover, different cell populations appear to be more or less sensitive to gene dose [8, 15]. These data are consistent with the hypothesis that, in the forebrain, MET signaling can influence developmental processes that underlie quantitative temporal and spatial aspects of connectivity. However, disruption in signaling alone is insufficient to create dramatic transformations such as those observed with genes that cause syndromic disorders that often are accompanied by intellectual disability and other severe impairments. Our hypothesis is also consistent with the recent discovery of a pedigree in which a heterozygous loss-of-function mutation of *MET* results in ASD or social-communication diagnosis, but no intellectual disability. Our current behavioral findings, subtle in nature, are also consistent with a modulatory role for *MET* gene dose in human brain growth (Hedrick, 2012). Human neuroimaging studies showed that the functional “C” promoter allele, which reduces gene transcription, correlates with reduced connectivity and functional activation of circuits when looking at emotional faces, even in the typically developing population [25].

The present study was the first attempt to reveal the behavioral impact of altering *Met* expression in the mouse. Unique to the study design was examination of the impact of integrated circuitry using two distinct driver lines to eliminate *Met* expression. We first eliminated *Met* using *Emx1*^*cre*^ to eliminate functional signaling from neurons derived from the dorsal pallium. These cells give rise in part to circuitry involved in mediating social and emotional behaviors disrupted in ASD. We also eliminated *Met* using the *Nestin*^*cre*^ driver, in which *Met* was deleted from all neural cells, thereby presumably impacting behaviors more globally, not only those considered core ASD behaviors. Because there are fundamental differences in the neocortical expression patterns of MET in primates compared to mice [10, 13, 14], with Met being more widespread in the rodent, it was not clear whether any core social-communication deficits that are associated with the C promoter allele would be altered in the mouse models. We therefore used a large repertoire of behavioral tasks, including basic motor function, probes of affective state, social proclivity, and complex learning that extend beyond mouse behaviors that have human correlates implicated in ASD. These additional behaviors, such as activity, anxiety, and attention, are not diagnostic of ASD but can be expressed by subgroups of children with the diagnosis [51–53].

In general, the *Met*^*fx*/*fx*^/*Emx1*^*cre*^ KO animals display hypoactivity across several behavioral tasks, but this does not appear to reflect impaired coordination, as their performance on the rotarod was significantly better than their WT littermates. Additionally, these animals display blunted spontaneous alternation, indicative of impaired spatial working memory. These animals show no differences in olfactory dishabituation, sociability, and social novelty preference, and their learning and retention of memory in fear conditioning was intact. The more global deletion of *Met* generated some surprising results but readily replicated in separate cohorts. First, the *Met*^*fx*/*+;fx*/*fx*^/*Nestin*^*cre*^ Het and KO lines do not express differences in locomotion or spontaneous alternation. In fact, the only behavioral task in which these animals differ from their WT littermates is contextual fear conditioning. Second, the more severe deficits measured in *Met*^*fx*/*+*^/*Nestin*^*cre*^ Het animals compared to the full KO or WT was unexpected. Het mice exhibited disruptions in both fear learning and memory, whereas the *Met*^*fx*/*fx*^/*Nestin*^*cre*^ KO animals demonstrate a disruption only in fear memory. While not statistically significant, the same trend for more disruptions in Het mice is present in both rotarod and activity chamber performance. As already noted, this was not seen with the heterozygous *Met*^*fx*/*+*^/*Emx1*^*cre*^ mice, as they do not show any behavioral differences from WT in the tasks used here (data not shown). Interestingly, Het and KO mice created with the same *Emx1*^*Cre*^ driver, however, do exhibit similar increases in excitatory drive on a subset of layer 5 pyramidal cells [15], suggesting that more targeted, advanced behavioral tasks that probe intracortical connectivity in mice would need to be used to demonstrate cellular and whole animal functional correlates.

**Fig. 7:**
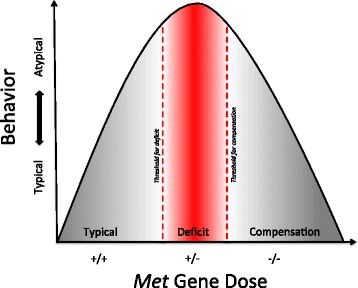
Theoretical model of *Met* gene dose effect on behavioral disruption. Our data indicate that intermediate expression of *Met* (*Met* ^*fx*/*+*^/*Nestin* ^*cre*^ Het) resulted in significant behavioral deficits while the absence of *Met* (*Met* ^*fx*/*fx*^/*Nestin* ^*cre*^ KO) only sometimes impacted behavior. The model suggests an inverse U-shaped model of atypical behavior such that an absence of *Met* can lead to compensatory changes in brain development. The model proposes a threshold of *Met* gene dosage for a behavior deficit and a second threshold of *Met* gene dosage that is permissive for compensatory mechanisms resulting in typical behavior

In contrast to the current report, most behavioral studies do not examine potential gene dose effects. When analyzed, Het animals typically are reported as not different from WT or intermediate between complete knockouts and controls [54–59]. There are several explanations for the more robust Het phenotype observed here. It is possible that *Met*^*fx*/*+*^/*Nestin*^*cre*^ Het mice, which express approximately 50 % of MET protein (Additional file 1), fail to exhibit compensatory mechanisms that may occur in complete *Met*^*fx*/*fx*^/*Nestin*^*cre*^ KO animals in which the early complete absence of MET signaling generates adaptations (Fig. 7). Certain experimental perturbations have revealed this phenomenon, such as the lack of phenotype when a gene is deleted genetically, but robust disruptions when expression is reduced partially in a subset of cells [60, 61]. In addition, even for genes that cause syndromic disorders, behavioral studies are complex due to issues of strain background, the genetic strategies for deletion, variation in testing environments across laboratories, and even testers [62–64].

Inverted “U” outcomes are common, in which the same physiological response or behavioral performance occurs with low or high amounts of hormones, neurotransmitters, or psychological stress [65–69]. Here, because the MET receptor tyrosine kinase converges on downstream intracellular systems (ERK and PI3 kinase) that mediate many receptor signaling cascades [70], it is possible that an imbalance in signaling during development is as, or more, detrimental than no signaling at all via this receptor. It is interesting that for ASD and intellectual disability, disruptions of the intracellular downstream components from multiple receptors are dominant [71, 72]. If such adaptive differences do occur, it is likely that there are cell type-specific effects (as seen electrophysiologically between neocortical [15] and CA1 hippocampal [8] pyramidal cells), because differences between WT and *Met*^*fx*/*+*^/*Emx1*^*cre*^ heterozygous mice were not obtained in the behavioral assays used here. It is important to note that these classic probes of behavior may not be sufficiently sensitive to detect differences in information processing due to altered cortical circuitry.

Finally, our studies of *Met/MET* to date have focused on understanding the role of the receptor tyrosine kinase itself in mediating typical behavioral performance. Studies utilizing ethologically relevant tasks that examine complex information processing may reveal further deficits. Additionally, a combination of genetic disruption and environmental factors will also need to be examined. For example, in humans, the *MET* functional C allele has been associated with environmental factors, such as ultrafine particle pollutants, that increase risk for ASD [22, 73, 74]. Thus, combining behavioral assays that target forebrain and hindbrain circuits, the latter being where *Met* is expressed in autonomic circuits prenatally [75], with human population-relevant environmental exposures may yield important avenues for discovering mechanisms of action.

## Additional file

Additional file 1:
**Met protein reduction in Met** ^**fx/+**^ **/Nestin** ^**cre**^ **heterozygous mice.** Representative blots of tissue homogenates for P21 frontal cortex were prepared for Western blots to examine MET expression according to Judson [11] with modifications. Note the reduction in the mature Met receptor (145 kd band) in Met^fx/+^/Nestin^cre^ Het mice compared to WT. Reduction was consistent and replicated in more than 10 mice per line.

## Abbreviations

AAR: alternate arm return
ANOVA: analysis of variance
ASD: autism spectrum disorder
CA1: cornu ammonis 1 region of hippocampus
Cm: centimeter
dB: decibel
EPM: elevated plus maze
ERK1: extracellular kinase 1
Het: heterozygous
kHz: kilohertz
KO: knockout
mA: milliamp
P: postnatal day
PI3: phosphoinositide 3
RPM: revolutions per minute
SA: spontaneous alternation
SAR: same arm return
WT: wildtype
